# Turning points in the evolution of peroxidase–catalase superfamily: molecular phylogeny of hybrid heme peroxidases

**DOI:** 10.1007/s00018-014-1643-y

**Published:** 2014-05-21

**Authors:** Marcel Zámocký, Bernhard Gasselhuber, Paul G. Furtmüller, Christian Obinger

**Affiliations:** 1 Division of Biochemistry, Department of Chemistry, VIBT, Vienna Institute of BioTechnology, BOKU, University of Natural Resources and Life Sciences, Muthgasse 18, 1190 Vienna, Austria; 2 Institute of Molecular Biology, Slovak Academy of Sciences, Dúbravská cesta 21, SK-84551 Bratislava, Slovakia

**Keywords:** Hybrid-type heme *b* peroxidase, Peroxidase–catalase superfamily, Molecular evolution, Multifunctional enzymes, WSC domain

## Abstract

Heme peroxidases and catalases are key enzymes of hydrogen peroxide metabolism and signaling. Here, the reconstruction of the molecular evolution of the peroxidase–catalase superfamily (annotated in pfam as PF00141) based on experimentally verified as well as numerous newly available genomic sequences is presented. The robust phylogenetic tree of this large enzyme superfamily was obtained from 490 full-length protein sequences. Besides already well-known families of heme *b* peroxidases arranged in three main structural classes, completely new (hybrid type) peroxidase families are described being located at the border of these classes as well as forming (so far missing) links between them. Hybrid-type A peroxidases represent a minor eukaryotic subfamily from Excavates, Stramenopiles and Rhizaria sharing enzymatic and structural features of ascorbate and cytochrome *c* peroxidases. Hybrid-type B peroxidases are shown to be spread exclusively among various fungi and evolved in parallel with peroxidases in land plants. In some ascomycetous hybrid-type B peroxidases, the peroxidase domain is fused to a carbohydrate binding (WSC) domain. Both here described hybrid-type peroxidase families represent important turning points in the complex evolution of the whole peroxidase–catalase superfamily. We present and discuss their phylogeny, sequence signatures and putative biological function.

## Introduction

Heme peroxidases are ubiquitous oxidoreductases present in all kingdoms of life. They can be divided in three main structural superfamilies and two minor families (overview in [1]). The three main superfamilies are the (1) peroxidase–catalase superfamily, (2) the peroxidase–cyclooxygenase superfamily [2] and (3) the CDE superfamily [3]. The latter is comprised of so-called *c*hlorite dismutases, *d*ye-decolorizing peroxidases and *E*feB (which are heme-binding/sensoring proteins of unclear biological function). In PeroxiBase (http://peroxibase.toulouse.inra.fr [4]) currently over 9,580 heme peroxidases are annotated and 8,160 of them belong to the peroxidase–catalase superfamily.

Heme peroxidases catalyze the oxidation of one- or two-electron donors by hydrogen peroxide. There is a great variability regarding the nature of one electron donors ranging from metal cations, aliphatic or aromatic compounds to proteins (Reaction 1). Typical two-electron donors can be halides (like Cl^−^, Br^−^ or I^−^) or thiocyanate (SCN^−^) which are oxidized to the corresponding hypohalous acids (HOX) (Reaction 2). There are only few heme peroxidase (sub) families that are able to dismutate hydrogen peroxide in the absence of electron donors as typical (monofunctional) catalases (Reaction 3). However, the mechanism of hydrogen peroxide dismutation by these peroxidases is different from typical heme catalases and thus should be designated as (pseudo) catalatic [5].

1 $${\text{H}}_{ 2} {\text{O}}_{ 2} \, + \, 2 {\text{AH}}_{ 2} \, \to \, 2 {\text{H}}_{ 2} {\text{O}}\, + \, 2 {\text{HA}}^{ \bullet }$$

2 $${\text{H}}_{ 2} {\text{O}}_{ 2} \, + \,{\text{X}}^{ - } \, + \,{\text{H}}^{ + } \, \to \,{\text{H}}_{ 2} {\text{O}}\, + \,{\text{HOX}}$$

3 $$2 {\text{H}}_{ 2} {\text{O}}_{ 2} \, \to \, 2 {\text{H}}_{ 2} {\text{O}}\, + \,{\text{O}}_{ 2}$$

This work focuses on the peroxidase–catalase superfamily (PF00141). Already in 1992, Welinder [6] for the first time recognized the phylogenetic and structural relationship between heme peroxidases from plants, fungi and bacteria. In the subsequent years, the denominations “plant-type peroxidase superfamily” or “superfamily of heme peroxidases from plants, fungi and bacteria” or “non-animal heme peroxidase superfamily” were used in literature. In the meanwhile numerous further representatives from the kingdoms of Archaea, Fungi, Protista and Plantae were detected. Importantly, even in some Metazoan genomes members of this superfamily were found. Thus, it is more appropriate to denominate the whole superfamily according to the typical reaction specificities of its members [1] and not according to the taxonomical origin of selected members (as usual in older literature, e.g., [7]. It will be outlined below, how bifunctional enzymes (i.e., catalase–peroxidases) form the basis of this superfamily and how during evolution the (pseudo-) catalatic activity was lost and monofunctional peroxidases evolved. The denomination peroxidase–catalase superfamily reflects this development. This superfamily represents a good example for the divergent evolution of a mid-size gene that acquired diverse functions in different genomes. The usage of manifold electron donors by the different organisms in Reactions 1 and 2 mirrors the physiological necessities. The particular peroxidase gene evolved either by vertical descent or by horizontal gene transfer (HGT) as observed in several clades within the evolutionary history of this superfamily [8].

All members of the peroxidase–catalase superfamily that were investigated at the protein level contain non-covalently bound heme *b* and have a histidine as proximal ligand as well as a catalytic distal histidine [9]. For all other residues in the heme cavity some variability is observed and described below. Three main classes are distinguished (Class I, II and III) of which many biochemical and physical data have been collected in the last decades (e.g., [10–12]). Only recently, a few of the evolutionary missing links between these classes started to be systematically investigated (e.g., [13]).

Class I of this superfamily (1,852 annotated members, classified according to [6] is apparently the most divergent one. It comprises sequences of various catalase–peroxidases, ascorbate peroxidases, cytochrome *c* peroxidases and also hybrid-type subfamilies discovered recently. Class II (609 annotated members) is constituted mainly by manganese, lignin and versatile peroxidases from lignin-degrading fungi [14], but also numerous novel heme peroxidase sequences from fungi, that are not able to decay wood, which were annotated recently [15]. Class III (5,701 annotated members) is formed by large multigenic families of plant secretory peroxidases [16] including the well-known horseradish peroxidase (HRP).

The intention of this paper is to highlight features of the missing links between these three main classes. Hybrid-type A peroxidases (also named ascorbate-cytochrome *c* peroxidases, APx-CcP) are shown to be positioned between the classical families of ascorbate and cytochrome *c* peroxidases, whereas hybrid-type B enzymes (originally also abbreviated as APx-CcPs) are quite different from hybrid-type A proteins. A distinct group of ascomycetous hybrid-type B enzymes are shown to be fusion proteins containing a N-terminal peroxidase domain and C-terminal WSC-carbohydrate binding domain(s). We discuss the evolution and typical sequence signatures for both hybrid peroxidases and speculate about their physiological function. It will be demonstrated that hybrid-type peroxidases represent real turning points in the robust evolution of three main structural classes of the peroxidase–catalase superfamily. Their future biochemical analysis will further contribute to the understanding of the evolution of structure–function relationships of these abundant oxidoreductases.

## Materials and methods

### Sequence data mining and multiple sequence alignment

All protein sequences used in this study were collected from PeroxiBase (http://peroxibase.toulouse.inra.fr [4]) where they were previously verified and annotated. Multiple sequence alignment of full-length protein sequences was performed with Muscle program [17] implemented in the MEGA 5 package. Optimized parameters were: gap open −2.9 gap extend 0, hydrophobicity multiplier 2. Maximum of alignment iterations was set to 1,000. Used clustering method was UPGMB for the first two iterations, for other iterations Neighbor-Joining (NJ) and minimal diagonal length 28. Obtained alignment was inspected mainly for the presence of conserved catalytic residues on both distal and proximal sides of the heme prosthetic group and further refined in GeneDoc [18]. Those sequences that did not possess the conserved essential residues in the heme cavity [i.e., distal Trp(Phe, Tyr)-His-Arg(Lys) and proximal His-Asp-Trp(Phe)] were segregated from the main alignment. After inspection and refinements the alignment used for phylogeny contained 490 full-length sequences. Sequences of observed peroxidase pseudogenes were analyzed separately. For this purpose a second independent alignment focused mainly on the non-functional ascorbate peroxidases was prepared with Muscle using the same parameters as above.

### Structural alignment

Structural neighbors within the peroxidase–catalase superfamily were identified with the Dali Database (http://ekhidna.biocenter.helsinki.fi/dali) where the PDB structural hits were sorted according to obtained Z-score [19]. Structural alignment was performed with ESPript program suite http://espript.ibcp.fr/ESPript/ESPript [20] where the input was the previously refined Muscle-alignment file. As top secondary structure of this alignment the sequence of *Burkholderia* KatG with known 3D structure was selected (PDB code 1MWV, Table 1). Parameters for similarity calculations were Risler, global score of 0.7, and consensus over 50 % were displayed. Obtained structural alignment was edited in GeneDoc [18].

**Table 1:** Overview on all known 3D structures of representatives of the peroxidase–catalase superfamily

Peroxidase abbrev.	Source	Class	PeroxiBase ID	PDB code
BpKatG	*B. pseudomallei*	I	2303	1MWV
EcoHPI––C domain	*E. coli*	I	2394	1U2J
HmaKatG1	*H. marismortui*	I	2440	1ITK
MtKatG1	*M. tuberculosis*	I	3551	1SJ2
SeKatG1	*S. elongatus* PCC7942	I	2426	1UB2
MagKatG2	*M. grisea*	I	2337	3UT2
PsAPx1	*P. sativum*	I	2462	1APx
NtAPx2	*N. tabacum*	I	3946	1IYN
GmAPx1	*G. max*	I	1954	1OAG
SceCcP	*S. cerevisiae*	I	2361	2CYP
LmAPx-CcP	*L. major*	I	2334	3RIV
ArMnP	*A. ramosus*	II	2404	1ARP
CcinPOX2a	*C. cinereus*	II	2403	1LYK
PcLiP5	*P. chrysosporium*	II	2409	1LLP
PcMnP1	*P. chrysosporium*	II	2379	1MNP
PerVP5	*P. eryngii*	II	2299	2BOQ
AhPrx4	*A. hypogaea*	III	102	1SCH
AruPrxC1A (HRP)	*A. rusticana*	III	90	1ATJ
Atprx53	*A. thaliana*	III	219	1PA2
GmPrx1	*G. max*	III	475	1FHF
HvPrx101	*H. vulgare*	III	68	1BGP
RrePrx01	*R. regia* (palm tree)	III	not yet	3HDL

The PeroxiBase ID and the corresponding PDB codes are given. Note that only structural data of wild-type proteins and not of variants or engineered mutants are presented

### Identification of introns and exons in putative peroxidase genes

For identification of donor splice sites and acceptor splice sites in various peroxidase genes the program suite NetAspGene 1.0 of the CBS server was used (http://www.cbs.dtu.dk/services/NetAspGene/). GT-AG consensus sequence for the borders between exons and introns was present in most, but not all hybrid-type peroxidase genes.

### Molecular phylogeny

Molecular phylogeny within the whole peroxidase–catalase superfamily was reconstructed using the MEGA package, version 5 [21]. Muscle-aligned protein sequences including sequences of all 22 proteins of this superfamily with known 3D structure were subjected to Neighbor-Joining (NJ) or Maximum-Likelihood (ML) methods. For NJ 1,000 bootstraps, Jones-Taylor-Thornton (JTT) model of distribution and γ parameter optimized to 0.88 was used. For ML 100 bootstraps, Whelan and Goldman (WAG) model of amino acid substitutions with 3 γ categories was applied. The branch swap filter was set to very strong and the number of threads was set to 1. Reconstructed rooted phylogenetic tree was depicted with the program FigTree (http://tree.bio.ed.ac.uk/) in a circular polar form with branches transformed as cladograms. The branching details for particular (sub) families were presented with the Tree Explorer program of the MEGA package in the rectangular form. Ancestral protein sequences were inferred from the ML-reconstructed tree using the ancestors option of the MEGA suite [21] and exported as current site ancestors and corresponding most probable sequences.

### Prediction of signal sequences and glycosylation in peroxidases

Putative signal sequences for selected peroxidase sequences were analyzed using the predictive algorithm of the program SignalP 4.1 (http://www.cbs.dtu.dk/services/SignalP/ [22]. The appropriate prediction database was chosen according to determined phylogenetic relationship. Intracellular sequences were further subjected to subcellular localization analysis using TargetP 1.1 from the same online suite [22]. The N- and O-glycosylation sites were predicted with NetNGlyc1.0 and NetOGlyc 4.0 servers, respectively [23].

### Homology modeling

Homology modeling of putative peroxidases from various subfamilies was performed with I-Tasser [24]. Obtained structural models were rendered with PyMOL (http://www.pymol.org).

## Results and discussion

### The peroxidase–catalase superfamily: division in three main classes

**Fig. 1:**
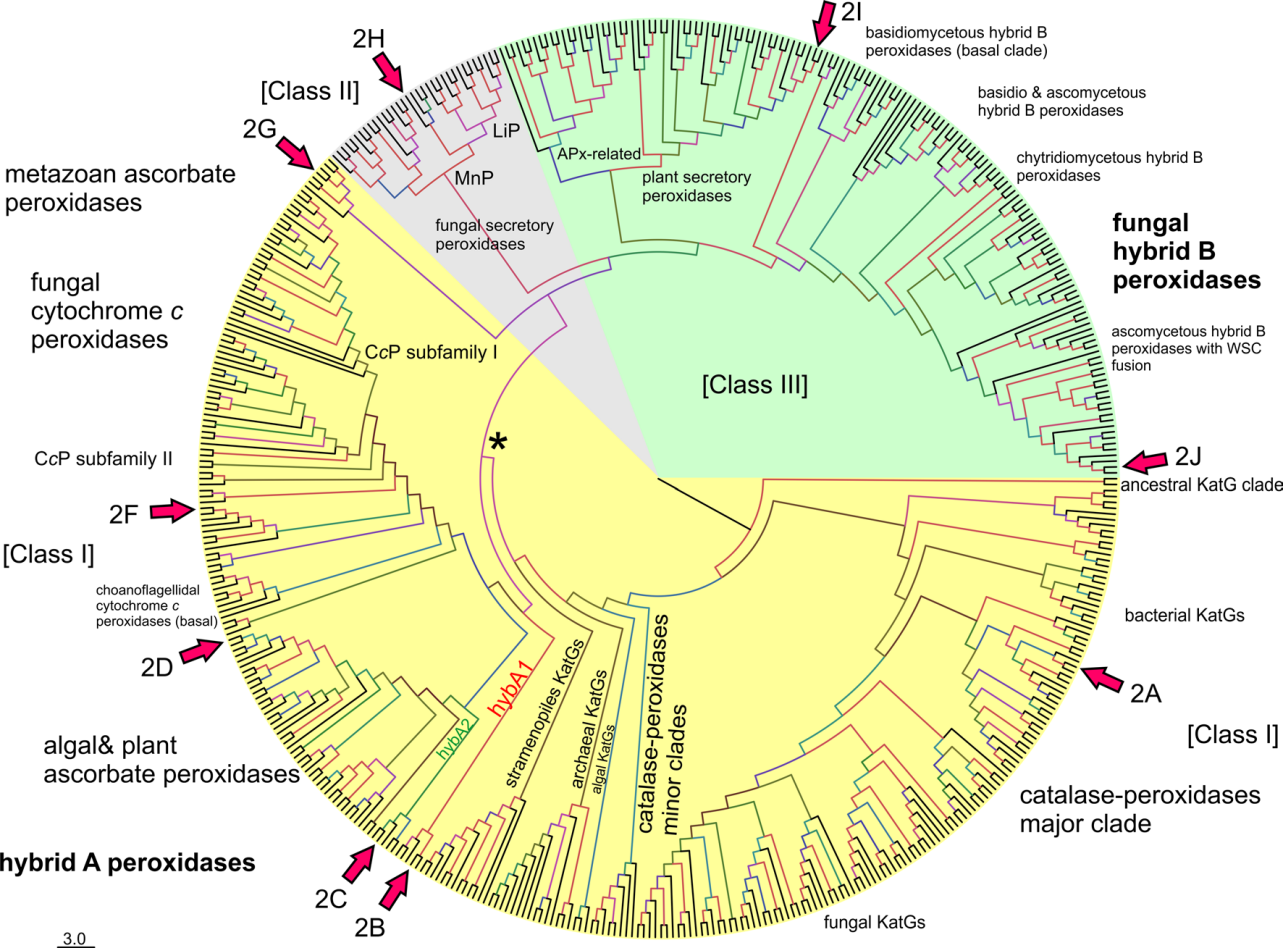
Rooted maximum-likelihood tree in a circular polar form reconstructed with 490 full-length sequences from the peroxidase–catalase superfamily. Bootstrap values are presented in a color scheme for the ML output: *red* >90, *violet* >70, *blue* >50, *green* >30. Three main classes and distinct subfamilies are highlighted. Node labeled as *Asterisk* is the evolutionary step where the two-domain structure and the bifunctionality were lost (see the discussion in the text). *Arrows* with number *2* and *alphabets* indicate the phylogenetic position of particular sequences analyzed in Fig. 2 (with exception of Fig. 2e which is a pseudogene)

We have reconstructed a robust phylogenetic tree of the whole peroxidase–catalase superfamily comprising up to 490 full-length protein sequences of members from all known subfamilies. The general presentation of this tree (Fig. 1) clearly distinguishes all three main structural classes already defined by Welinder in 1992 [6]. However, upon closer inspection the occurrence of many so far undescribed clades as well as missing links is obvious.

As expected, Class II and Class III (comprising only eukaryotic proteins) are evolutionary descendants of Class I, which contains both prokaryotic and eukaryotic representatives. The phylogenetic origin of Class I is (according to the ML method) positioned among predecessors of *katG* genes coding for catalase–peroxidases (KatGs) from planktonic aerobic and heterotrophic bacteria [25]. Catalase–peroxidases are homodimeric oxidoreductases having a two-domain monomeric structure with a functional N-terminal heme-containing domain and a C-terminal domain without prosthetic group. KatGs are bifunctional having both a (pseudo-) catalase activity [5] and a broad peroxidase activity [26, 27]. From the planctobacterial branch, a major KatG clade [28] and minor KatG branches evolved (Fig. 1). The major KatG clade includes sequences from all bacterial phyla but also fungal *katG* genes. Among fungal KatGs the majority is located intracellularly (either in the cytosol or in peroxisomes) [29]. A smaller second group contains signal sequences for secretion [8]. The further evolutionary development of the catalase–peroxidase superfamily led towards (so far) putative KatGs from eukaryotic photosynthetic Algae and heterotrophic Stramenopiles positioned still within the minor KatG branches.

**Fig. 2:**
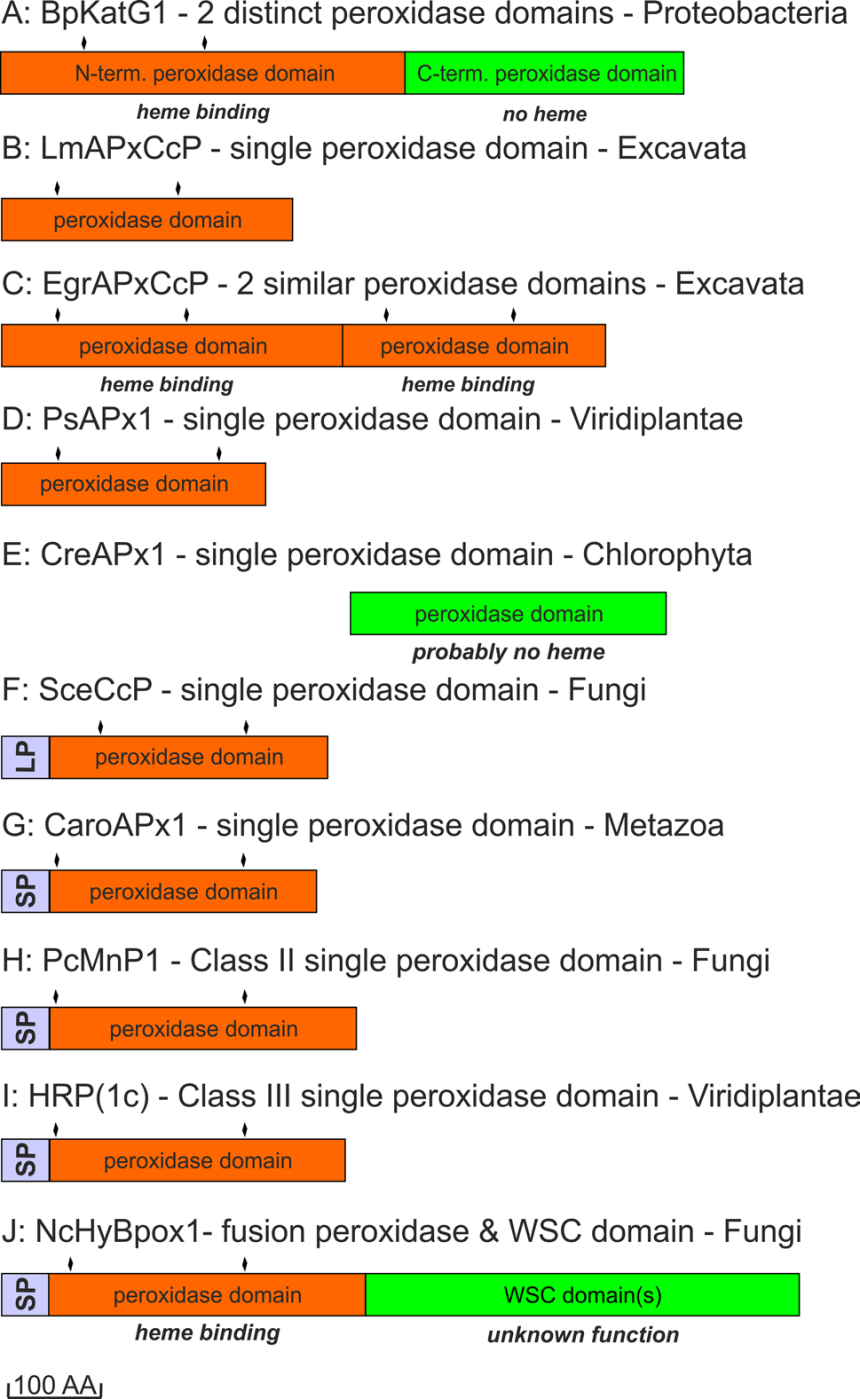
Schematic presentation of monomer organization in typical representatives of various classes of the peroxidase–catalase superfamily. *LP* leader peptide, *SP* signal peptide, *WSC* carbohydrate binding domain (for *w*ater-*s*oluble *c*arbohydrates), *aa* amino acid. Abbreviations of peroxidase names correspond with PeroxiBase. *Rhomboids* indicate positions of distal and proximal histidines

The next important evolutionary branching beyond bifunctional KatGs proceeded first in direction of hybrid-type A1 peroxidases that formed the basis for the evolution of three important groups within Class I, namely minor hybrid-type clade A2, ascorbate peroxidases from Algae and green plants and cytochrome *c* peroxidases from Opisthokonts phyla Choanoflagellida and Fungi (Fig. 1). Importantly, very soon in the evolution of the catalase–peroxidase superfamily (but already in the early eukaryotic world) at the beginning of the evolution of hybrid-type A peroxidase a common ancestor (labeled as * in Fig. 1) of fungal Class II peroxidases (including manganese and lignin peroxidases) and recently discovered and rare metazoan ascorbate peroxidases as well as of Class III peroxidases segregated. Most probably at this stage the two-domain structure typical for ancestral and modern KatGs disappeared by losing the (heme-free) domain (Fig. 2).

Besides KatGs only very few representatives of Class I are composed of two domains (Fig. 2c). Inspection of sequence suggests that in these rare cases both domains most probably are able to bind the prosthetic group (see following discussion of hybrid-type A peroxidases). There are also some examples of the occurrence of peroxidase-like domains lacking both the distal and the proximal histidines and thus (most probably) the heme group (Fig. 2e).

**Fig. 3:**
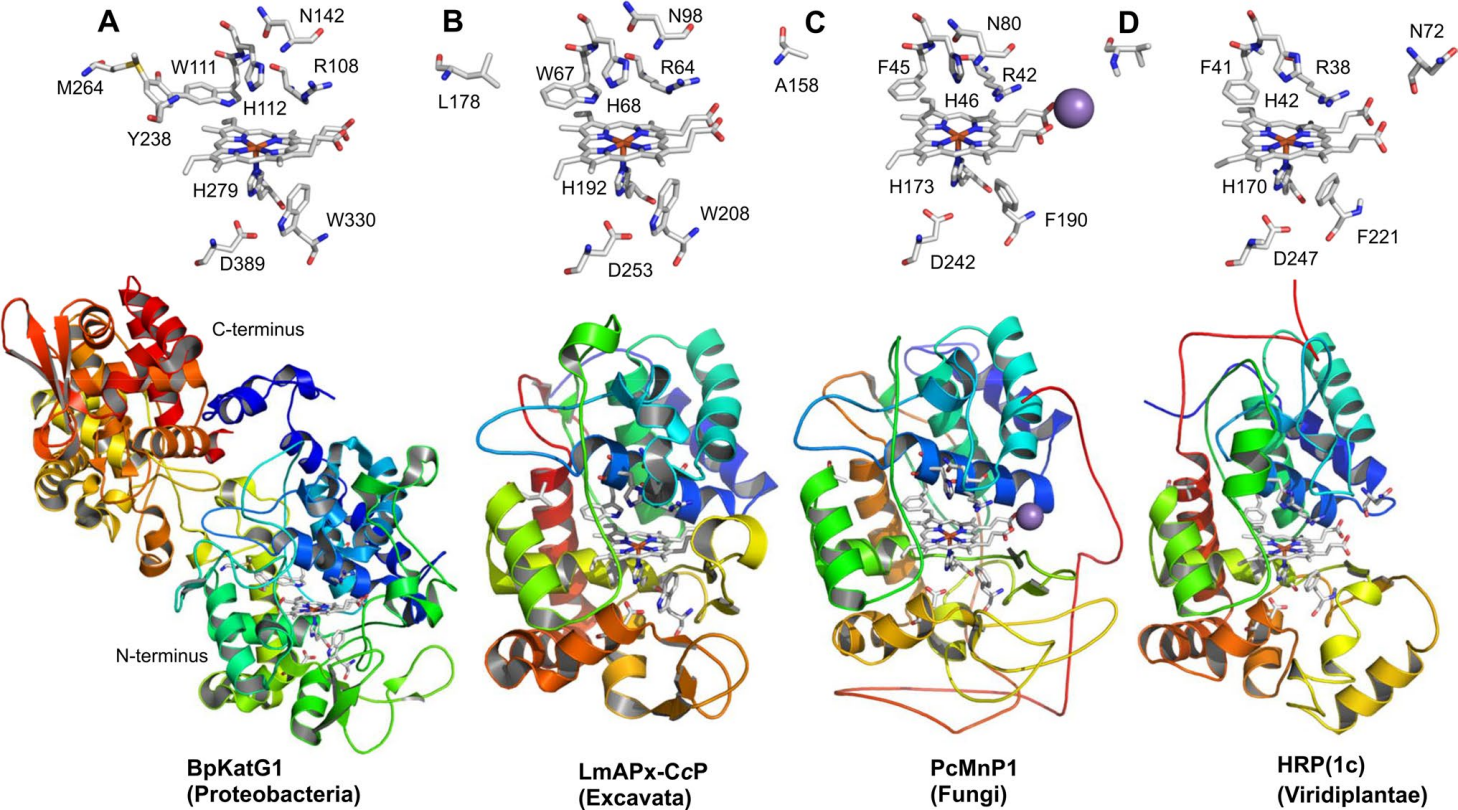
Three-dimensional structures of representatives from all classes of the peroxidase–catalase superfamily. **a**, **b** Class I peroxidases, **c** Class II and **d** Class III peroxidases. **a** Two-domain monomeric structure of KatG from *Burkholderia pseudomallei* (PDB accession code: 1MWV, **b** hybrid-type A1 peroxidase from *Leishmania major* (PDB code: 3RIV), **c** manganese peroxidase from *Phanerochaete chrysosporium* (PDB code: 1MNP) and **d** *Armoracia rusticana* (horseradish) peroxidase (PDB code: 1ATJ). Color code: N-terminus is depicted in *blue*, C-terminus in *red*. In addition, the heme *b* group and essential distal and proximal residues are depicted. The *violet* sphere in (**c**) represents a bound manganese cation. Abbreviations of peroxidase names are taken from PeroxiBase

More typically, most known ascorbate peroxidases (APxs) (Fig. 2b, d) and all known cytochrome *c* peroxidases (CcPs) (Fig. 2f) are single domain proteins having lost the ancestral C-terminal domain. This is also true for all currently known members of Class II and Class III (Fig. 2g–j) that reveal a high overall sequence similarity with the N-terminal domain of the ancestral KatG. The high level of evolutionary conservation of the heme peroxidase domain is obvious by comparison of the available X-ray structures (Table 1) of selected members as depicted in Fig. 3. The typical conserved 12-α-helical bundle [30, 31] with low content of β-strands did not change significantly during evolution.

### Peculiarities of hybrid-type A peroxidases

**Fig. 4:**
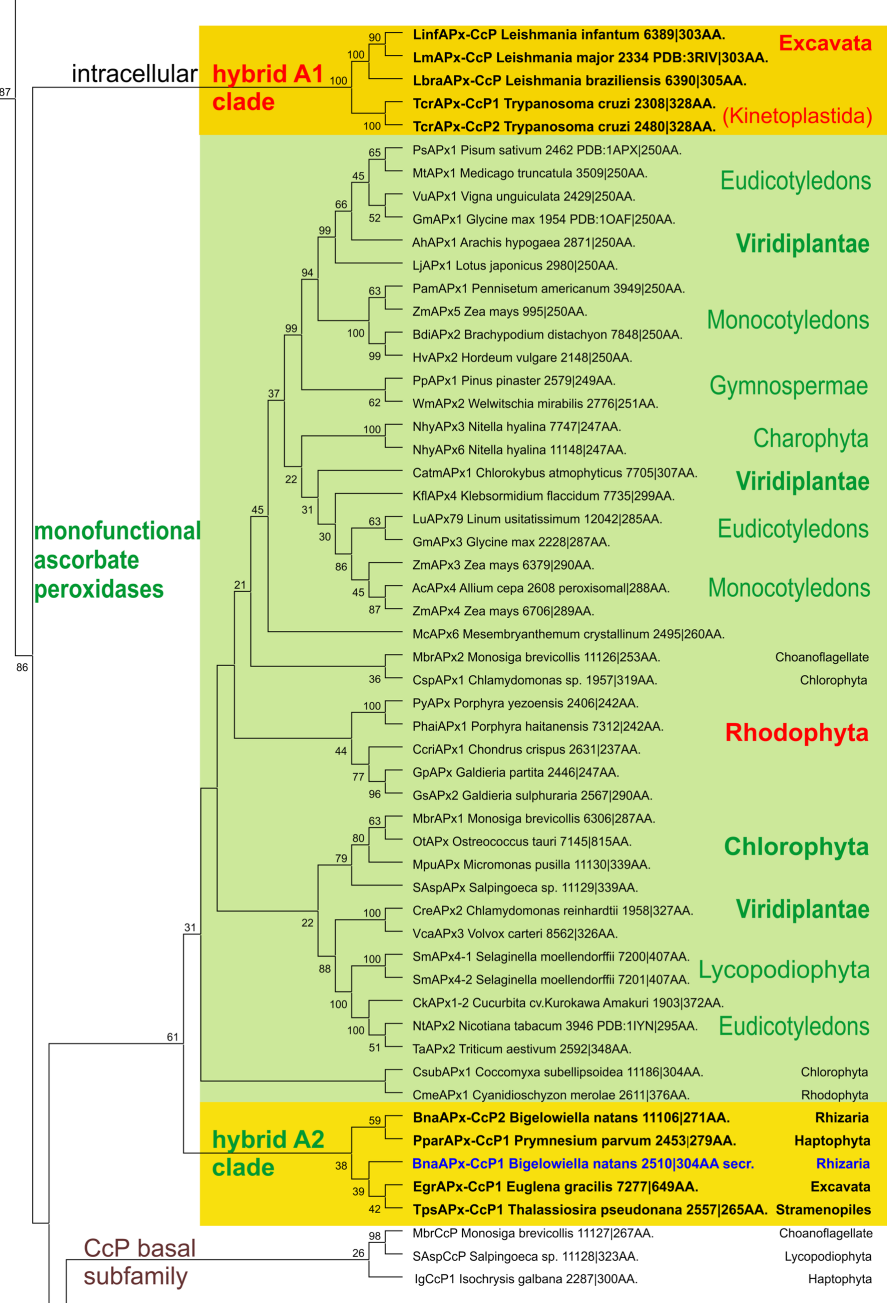
Details of the phylogenetic tree (Fig. 1) focusing on the hybrid-type A peroxidase clades. Obtained bootstrap values are presented for the ML method. Abbreviations of peroxidase names and corresponding ID numbers are taken from PeroxiBase. Subcellular location of hybrid A peroxidases is indicated

The investigation of hybrid-type A peroxidases (abbreviated as APx-CcP in PeroxiBase) started recently since it was found that they represent the missing link between ascorbate and cytochrome *c* peroxidases (Fig. 1). The presented detailed phylogenetic reconstruction (Fig. 4) additionally underlines that hybrid-type A peroxidases are also among first descendants of bifunctional catalase–peroxidases. Upon losing the (KatG-typical) C-terminal domain as well as the ability to dismutate hydrogen peroxide they became monofunctional peroxidases. It has been demonstrated frequently that ancient enzymes were promiscuous and thus multifunctional before evolving more specialized catalytic functions later during evolution [32].

**Fig. 5:**
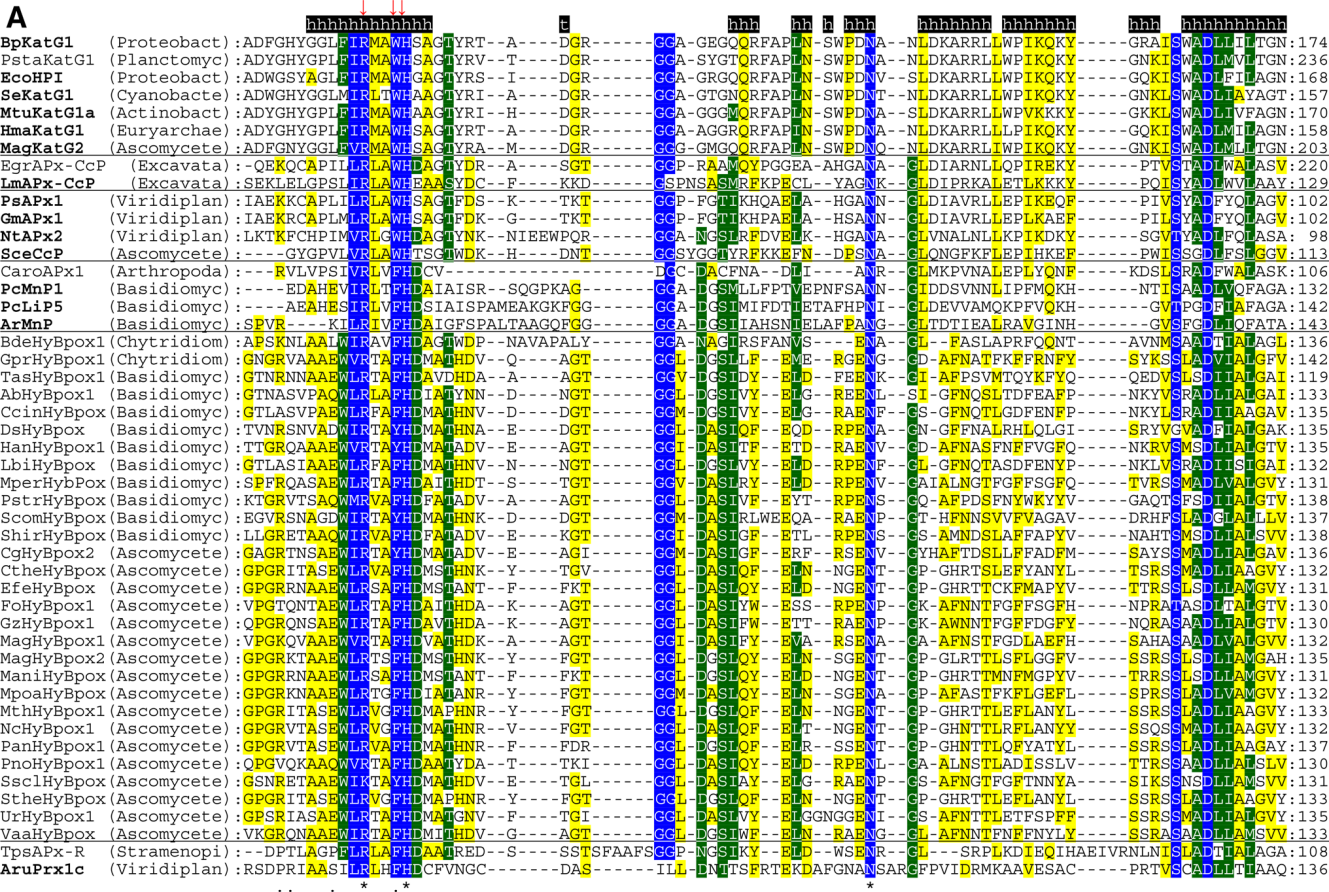

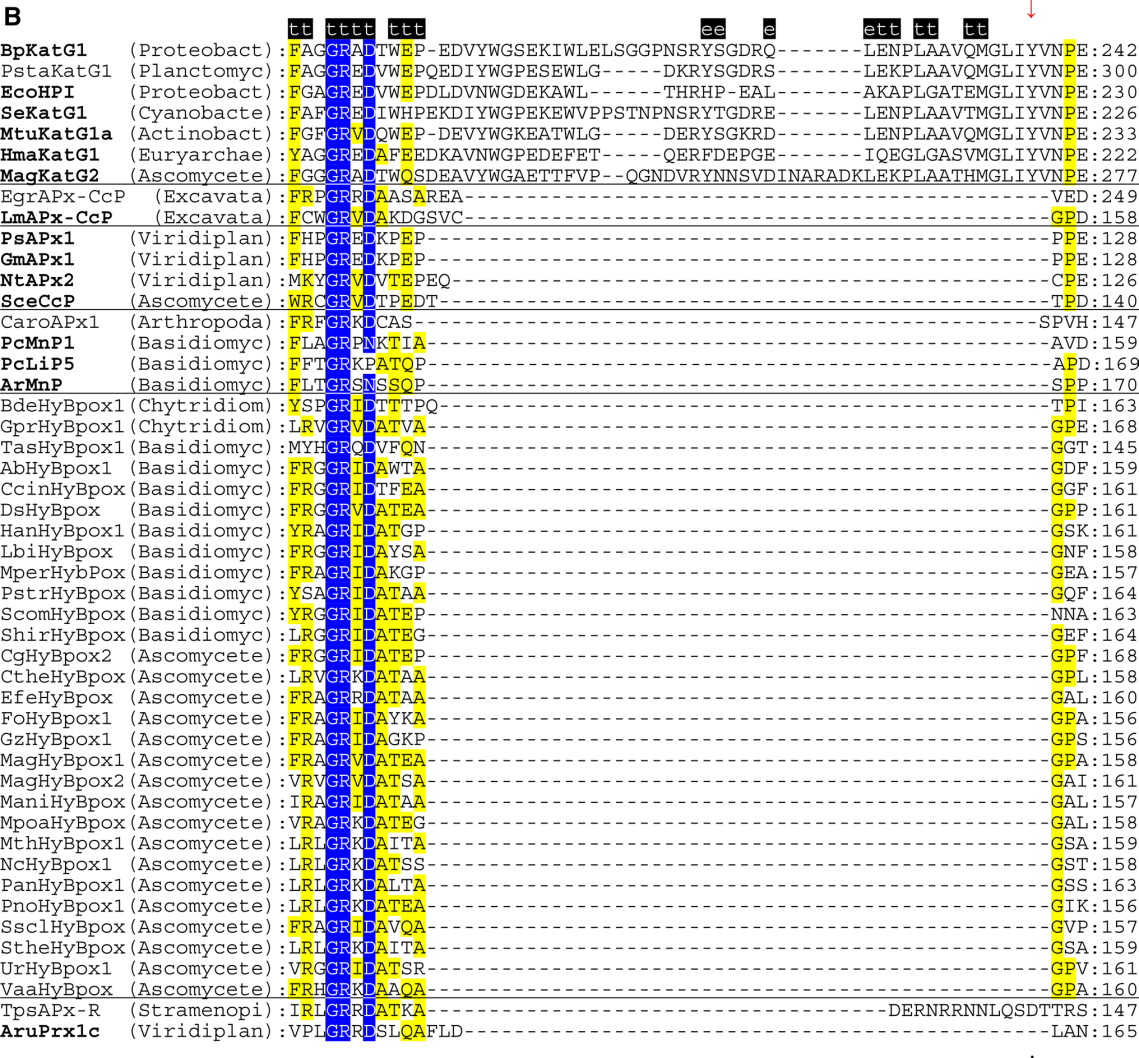

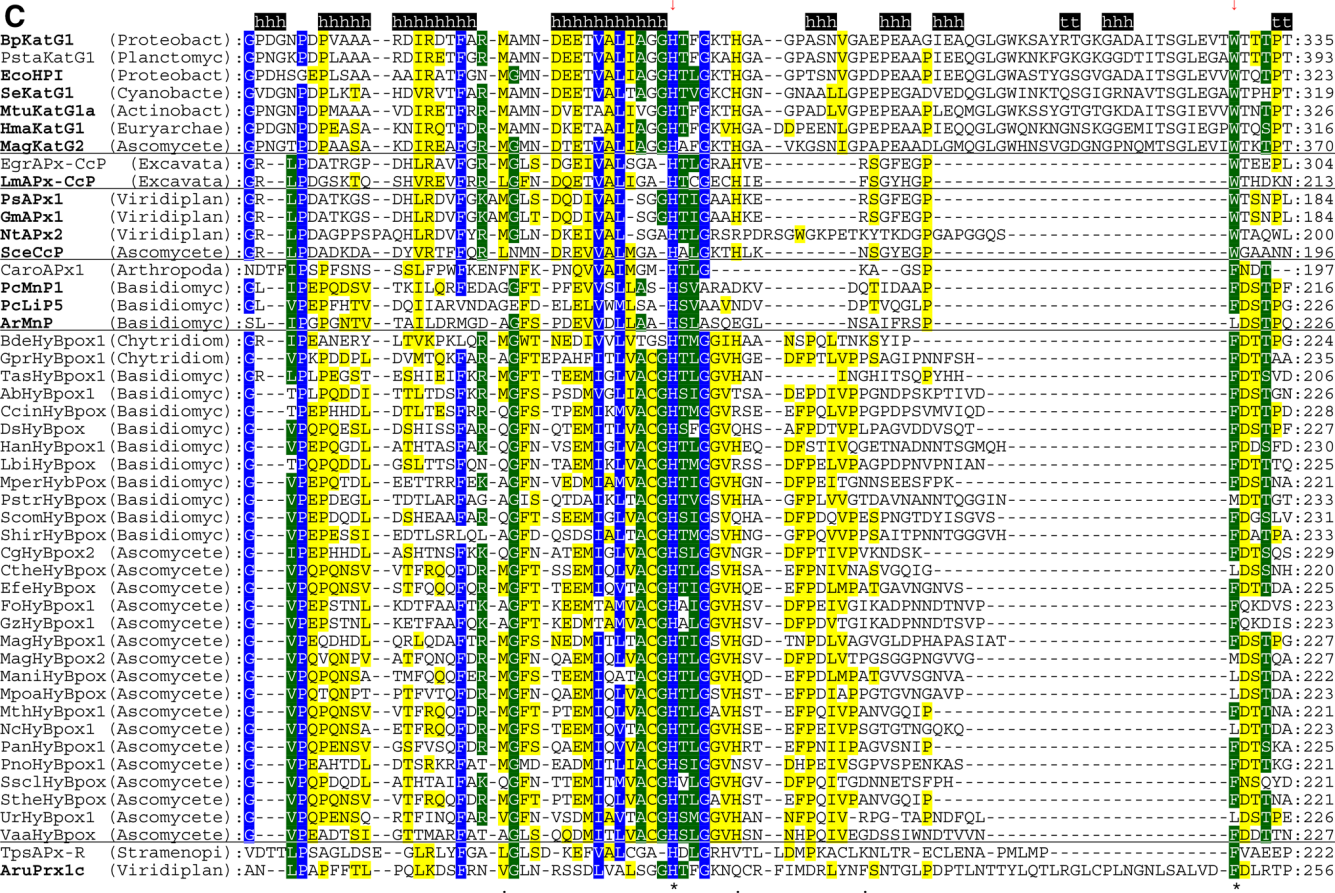
Selected parts of structural multiple sequence alignment of 48 members of the peroxidase–catalase superfamily. This alignment demonstrates both high conservation of the active site residues as well as some variability. **a** Region including residues at the distal heme side, **b** region of the large loop, **c** region including residues at the proximal heme side. Secondary structural elements taken from the 3D structure of KatG from *Burkholderia pseudomallei* (BpKatG, PDB code 1MWV) are depicted (*h* helix, *e* strand, *t* turn). Essential residues involved in catalysis are labeled as “*” and those residues that were involved in catalysis but later during the evolution mutated as “.” residues discussed in the text are labeled with *arrows*. Sequences with known 3D structures are in bold. Parameters for the alignment are described in the Sect. “Materials and methods”. Abbreviations of peroxidase names are taken from PeroxiBase

The multiple structural sequence alignment depicted in Fig. 5 suggests that the bifunctional activity of KatGs [5] has been lost stepwise. This can be exemplified by inspection of two representative hybrid-type A peroxidases, namely EgrAPx-CcP and LmAPx-CcP (Fig. 5). The (pseudo-) catalase activity of KatGs is based on the presence of a redox active cofactor, a post-translationally formed Trp-Tyr-Met adduct in close proximity to the heme group (BpKatG numbering: Trp111-Tyr238-Met264, Fig. 3a) [33]. Tyrosine 238, which is essential for the H_2_O_2_-degrading activity of KatG [34, 35], is located on the KatG-typical large loop LL1 that also contributes to the architecture of the substrate channel [5]. Both hybrid-type A peroxidases, EgrAPx-CcP and LmApx-CcP, have lost this large loop including Tyr238 (Fig. 5b) as well as the C-terminal part of large loop LL2 (Fig. 5c). Thus, they are not able to form the covalent adduct [10]. In LmAPx-CcP Met264 is substituted by a leucine, whereas in all hybrid-type A peroxidases Trp111 is fully conserved together with the catalytic residues His112 and Arg108 (BpKatG numbering Fig. 5). The histidine–arginine pair is found in all (mono) functional peroxidases of the catalase–peroxidase superfamily and is important for the heterolytic cleavage of H_2_O_2_ in compound I formation [36]. The proximal heme architecture including the triad His279––Asp389––Trp330 (and the H-bonding network between these residues) of hybrid-type A peroxidases is still very similar to that of KatGs [37].

Hybrid-type A1 peroxidase from *Leishmania major* has been investigated in detail demonstrating that it can use both ascorbate (as monofunctional APx) and cytochrome *c* (as monofunctional CcP) as electron donors (Reaction 2) [38]. The elucidation of its X-ray structure [30] confirmed its intermediate position between ascorbate and cytochrome *c* peroxidases (see Fig. 3b and discussion below).

Hybrid-type A heme peroxidases form a small group within lower single cell eukaryotes (Fig. 1). Phylogenetically, they can be subdivided in the basal subfamily A1 from Trypanosomatids and subfamily A2 from Rhizaria and Excavates (Fig. 4). The latter evolved in parallel with monofunctional ascorbate peroxidases. Sequence analysis suggests that hybrid-type A peroxidases are mostly non-secretory oxidoreductases which is reminiscent of most bacterial KatGs which are their predecessors. Only the circozoan *Bigelowiella natans* reveals two paralogs with one being presumably secreted to cope with environmental stress (highlighted in blue in Fig. 4). This marine alga of secondary endosymbiotic origin contains a battery of oxidoreductases all possessing signal/leader peptides [39].

Among hybrid-type A2 peroxidases a unique dimeric two-domain structure is found in the peroxidase of *Euglena gracilis* (EgrAPx-CcP1). At first sight, this resembles the two-domain structure of KatGs (Figs. 2a and 3a), but it has been demonstrated that both domains bind heme and exhibit peroxidase activity [40]. This two-domain peroxidase is localized in the cytosol of *Euglena gracilis* [40]. All other currently known hybrid-type A peroxidases do not contain a second heme-binding peroxidase domain.

### Large diversity within clades of ascorbate and cytochrome *c* peroxidases

As outlined above, ascorbate peroxidases are evolutionary descendants of hybrid-type A1 peroxidases (Fig. 4). The main physiological role of modern APxs is hydrogen peroxide reduction by concomitant oxidation of ascorbate to monodehydroascorbate according to Reaction 1. The early segregating APx clade containing only microalgal representatives is strictly intracellular as are their direct predecessors. In the descendant clades from green and red alga and higher plants most representatives are located either in the cytosol or in the plastids, but few members reveal also a peroxisomal location (e.g., AcAPx4 and ZmApx4) [41]. As monodehydroascorbate reductase is also partially localized in protistan and plant peroxisomes [42] this organelle could also possess an efficient ascorbate-recycling system.

The physiological role of ascorbate from cyanobacteria through alga towards higher plants has undergone an interesting stepwise evolution serving not only as a major cellular water-soluble antioxidant and cofactor for many enzymes but also as a growth regulator and signal transducer [43]. The significant higher concentration of ascorbate in plants compared to cyanobacteria [44] was directly connected with the amplification of genes encoding both APxs (cf. PeroxiBase) and dehydroascorbate reductases [42]. Ascorbate peroxidases have neither a reasonable H_2_O_2_ dismutating activity (like KatG) nor can they use cytochrome *c* as electron donor (as CcP). The substrate ascorbate binds at the γ-heme edge through hydrogen bonds to conserved basic residues (typically lysine and arginine) and a heme propionate [45]. The proximal tryptophan (of the His-Asp-Trp triad) is (in contrast to CcP) not redox active [46]. During evolution of APx the distal tryptophan was changed to phenylalanine (as found in most Class II and Class III peroxidases).

**Fig. 6:**
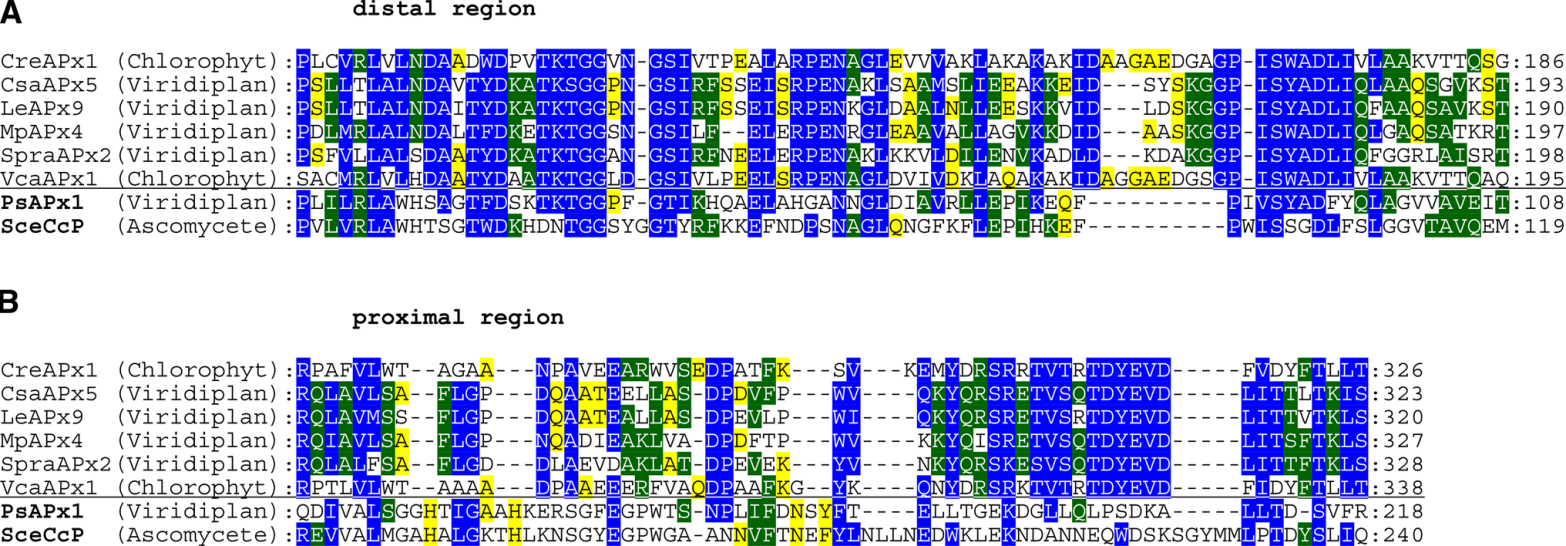
Multiple sequence alignment presenting peroxidase-like genes coding for variants of plant ascorbate peroxidase-like proteins that most probably lack the heme group. Sequences are compared with functional ascorbate peroxidases from *P. sativum* (PsAPx) and yeast cytochrome *c* peroxidase (SceCcP) with known 3D structures. **a** Region of residues around distal histidine. **b** Region of residues around proximal histidine. Parameters for the alignment are described in the Sect. “Materials and methods”. Abbreviations of peroxidase names are taken from PeroxiBase

The present analysis has also shown that several APx genes in Chlorophyta and higher plants lack most of the essential residues for heme binding and/or catalysis (Fig. 6a, b). This is in contrast to the hybrid-type A2 peroxidase from *Euglena gracilis* (EgrAPx-CcP1) with its unique structure of two functional domains [40]. This leads us to the hypothesis that right at the level of segregation from bifunctional KatGs to monofunctional peroxidases (labeled with * in Fig. 1) the two-domain version might have existed for some time before being separated and loosing functionality (Fig. 6). The occurrence of separated (heme-free) KatG-like C-terminal domains still present in some genomes (cf. PeroxiBase) supports this hypothesis.

Also monofunctional cytochrome *c* peroxidases are direct descendants of hybrid-type A peroxidases (Fig. 1). Phylogenetic analysis suggested the presence of two CcP subfamilies. At the beginning of CcP evolution choanoflagellidal enzymes are found (Figs. 1 and 4). As expected from their direct predecessors they are intracellular enzymes and some of them are predicted (with high probability) to be targeted to mitochondria. Also the newly discovered Stramenopiles CcP representatives like most of the fungal CcPs of subfamily II [47] could be targeted to mitochondria. By contrast, CcP subfamily I [47] was predicted as non-mitochondrial, probably cytosolic. As obvious from here presented phylogenetic reconstruction this younger subfamily could have evolved through the loss or modification of the ancestral leader sequence.

In both CcP subfamilies the architecture of the heme cavity seems to be well conserved (i.e., distal triad Trp-His-Arg and proximal triad His-Asp-Trp). Cytochrome *c* peroxidases can neither dismutate H_2_O_2_ nor use ascorbate as electron donor. In contrast to APx the proximal Trp (Trp191 in SceCcP) is the site of entry for both cytochrome *c* electrons when CcP follows Reaction 1. The physiological role(s) of CcP is still under discussion. The enzyme from *S. cerevisiae* seems to be a protective enzyme for aerobic metabolism and is localized in the intermembrane space of mitochondria where it consumes hydrogen peroxide generated in the respiratory electron transport chain [48]. The physiological role of cytosolic CcPs of subfamily I remains unclear.

### Metazoan representatives of the peroxidase–catalase superfamily

Originally this superfamily was always designated as “non animal” or as “superfamily of plant, bacterial and fungal peroxidases” [6, 7]. Our analysis clearly demonstrates that a distinct clade of (putative) ascorbate peroxidases was segregated very early in evolution (Fig. 1). It contains only sequences from Unikonts/Metazoan lineages. Already described symbiotic *Hydra viridis* ascorbate peroxidase [49] belongs to this clade in addition to other sequences of metazoan origin. Most of them are predicted as secretable proteins (Table 2). Since all descendant clades including Class II, Class III and hybrid-type B peroxidases (Fig. 1) also contain signal sequences for secretion, this might reflect an evolutionary turning point with respect to subcellular targeting. In case of the marine sponges from the phylum Porifera secretable peroxidases could help to combat marine microbes that live in the same niche [50]. In sea lice (i.e., copepod parasites of fish) these peroxidases might be involved in coping with the immune defence reactions of the host [51]. Whether ascorbate is the preferred electron donor of peroxidases from this minor clade remains to be verified experimentally as well as the eventual physiological role of ascorbate in ancestral metazoan lineages.

**Table 2:** Prediction of signal and leader peptides in various (sub) classes of the peroxidase–catalase superfamily performed with SignalP (http://www.cbs.dtu.dk/services/SignalP/) or TargetP (http://www.cbs.dtu.dk/services/TargetP/)

Peroxidase	PeroxiBase ID	Taxonomy	Signal or leader sequence length AA, (probability)
AqAPx	11256	Porifera	S 19 (0.959)
AbHyBpox1	7607	Basidiomycete	21 (0.592)
BnaAPx-CcP1	2510	Cercozoa	S 22 (0.964)
BnaAPx-CcP2	11106	Cercozoa	cytosolic (0.916)
CaroAPx1	7150	Ecdysozoa	S 17 (0.959)
CgHyBpox1	5357	Ascomycete	S 21 (0.646)
CgHyBpox2	5361	Ascomycete	S 22 (0.872)
CtheHyBpox	10136	Ascomycete	S 20 (0.560)
GprHyBpox1	11620	Chytridiomycete	S 29 (0.652)
MagHyBpox1	2621	Ascomycete	S 19 (0.683)
MagHyBpox2	5356	Ascomycete	S 22 (0.836)
NcHyBpox1	5358	Ascomycete	S 20 (0.589)
TasHyBpox1	11638	Basidiomycete	S 20 (0.810)
TcrAPx-CcP1	2308	Kinetoplastida	Mt (0.881)

Abbreviations of used sequence names and ID correspond to PeroxiBase

*S* secreted, *Mt* mitochondrial

### Peculiarities of hybrid-type B peroxidases

Until now hybrid-type B peroxidases have been abbreviated as “APx-CcP” similar to their hybrid-type A counterparts. However, it is obvious from sequence alignment (Fig. 5c) and first available experimental data (unpublished) that they are not able to use cytochrome *c* as electron donor. Their phylogenetic position is very far from both clades of APxs and CcPs (Fig. 1). Moreover, the basic residues responsible for ascorbate binding in APx at the γ-edge of heme [12] have partially been lost on the distal, but mainly on the proximal side (Fig. 5a, c). At the same time they have acidic Mn^2+^-binding residues on the distal side (Fig. 5a) which are conserved in all Class II manganese peroxidases [52]. The proximal heme cavity residues are identical to those from Class III peroxidases (Fig. 5c).

An interesting variability in the peroxidase active site among hybrid-type B occurs in the distal catalytic triad (Fig. 5a). Instead of the usual, highly conserved motif “Arg-X-X-Phe/Trp-His” “Arg-X-X-Tyr-His” is found in several proteins, e.g., in basidiomycetous DsHyBpox, HanHyBpox, MperHyBpox, ScomHyBpox1 and ascomycetous CgHyBpox2. Moreover, “Lys-X-X-Tyr-His” is found in phytopathogenic *Sclerotinia sclerotiorum* hybrid-type B peroxidase. Whether the modification of distal Trp/Phe towards Tyr affects the catalytic properties has to be tested. Whether a distal lysine might replace the arginine during heterolytic cleavage of hydrogen peroxide has to be tested too [53].

The present phylogenetic reconstruction demonstrates that hybrid-type B peroxidases are a sister clade to Class III and APx-related peroxidases (Fig. 1). We suggest to use the extension HyBpox for all members of this subfamily in future. The evolution of Class III peroxidases (including the well-known horseradish peroxidase, HRP) was reconstructed recently [54]. The main conclusion was that the emergence of Class III peroxidases was connected with the appearance of land plants as there were no members of this otherwise abundant Class found among Chlorophyta (green alga) so far. However, the present robust phylogenetic reconstruction points to a more complex scenario (Fig. 1). There was a common ancestor of hybrid-type B, APx-related and Class III peroxidases. Hybrid-type B peroxidases diverted early from the common node with Class III and APx-related (putative) peroxidases. The latter were separated later in evolution from the very abundant Class III clade (so far more than 5,700 sequences of Class III peroxidases are deposited in PeroxiBase). Interestingly, APx-related enzymes, which appear also in Bacillariophyta and Chlorophyta, have lost their ability to duplicate [55, 56]. This is in large contrast with typical Class III peroxidases genes present only among Viridiplantae that underwent frequent duplication events, giving rise to often more than 150 unique peroxidase genes in a single plant genome (e.g., *Zea mays* cf. PeroxiBase).

**Fig. 7:**
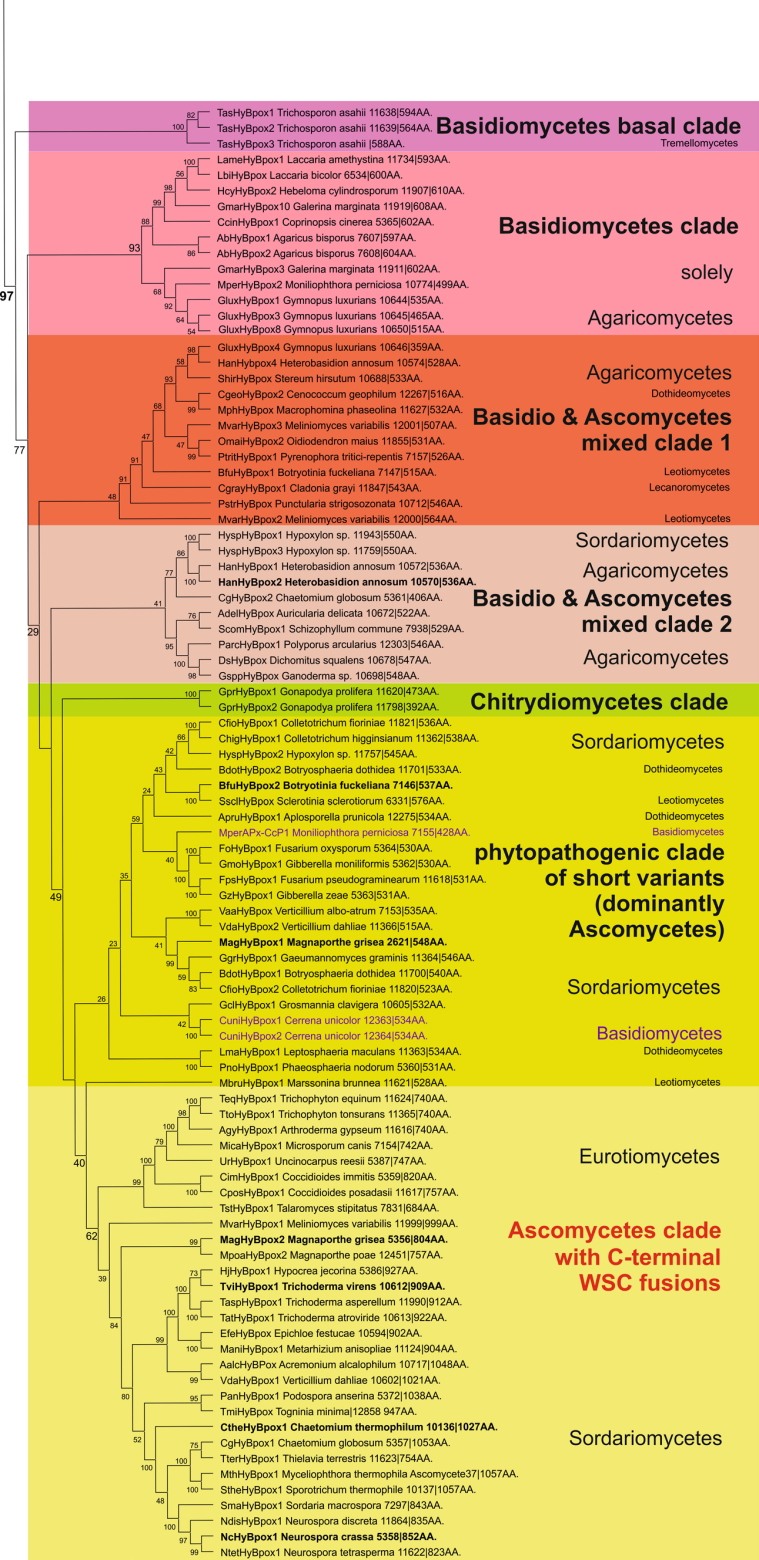
Details of phylogenetic tree (Fig. 1) of hybrid-type B peroxidases. Obtained bootstrap values are presented for ML method. Abbreviations of peroxidase names and corresponding ID numbers are taken from PeroxiBase. Those sequences where mRNA was detected (EST database) are labeled in bold

Hybrid-type B peroxidases are found in all fungal phyla, predominantly in phytopathogens. Their evolution occurred parallel with that of Class III peroxidases of land plants. Up to seven distinct clades of hybrid-type B peroxidases (Figs. 1 and 7) can be distinguished. In the basal clade for the whole hybrid-type B subfamily an enzyme from the basidiomycete *Trichosporon asahii* is found, which is a yeast-like fungus commonly inhabiting soils. It was described as an opportunistic human pathogen [57].

The next descendant clade contains only sequences from Agaricomycetes. So far among Agaricomycetes the expression of Class II peroxidases involved in lignin degradation was studied intensively [14], the physiological role of the hybrid-type B peroxidases is fully unknown. Interestingly, up to ten gene duplicates of hybrid-type B are found in a single fungal genome (e.g., in *Gymnopus luxurians* or *Galerina marginata,* cf. PeroxiBase). Thus, *hyBpox* gene behaves similar to Class III peroxidase genes in particular genomes.

The next two clades (Fig. 7) comprise genes from both Basidiomycetes and Ascomycetes. Thus, a lateral gene transfer between different fungal phyla cannot be ruled out so far. We have already an evidence from the EST database (Table 3) that this type of peroxidase is expressed in the basidiomycetous forest pathogen *Heterobasidion annosum*. A more detailed inspection of the genomic and transcriptomic context of these hybrid peroxidase genes is needed. Genes from the aquatic plant degradative fungus *Gonapodya prolifera* can be found in the small Chytridiomycete clade. Even more surprising is the position of another chytrid peroxidase from the amphibian pathogen *Batrachochytrium dendrobatidis.* It is closely related to Cryptogam Class III peroxidases and basal for all APx-related genes. The possibility of horizontal gene transfer of the hybrid-type B peroxidase genes into and from Chytridiomycota genomes is probable. However, so far only a few genes from this ancestral (and rather rare) phylum are available to answer this question satisfactorily.

**Table 3:** Expressed sequence tags with fragments of hybrid-type B peroxidase genes

EST #	Peroxidase	Source	Length [bp]	Remark
FQ924977	BfuHyBpox2	*B. fuckeliana*	665	Xylan as carbon source
JZ584098	CtheHyBpox	*C. thermophilum*	397	Mycelium induced with H_2_O_2_
CCOZ4053	HanHyBpox2	*H. annosum*	789	Culture from liquid Hagem medium
CD037344	MagHyBpox1	*M. grisea*	642	Subtracted mycelial library
DC977217	MagHyBpox2	*M. grisea*	366	Growth in nitrogen limiting conditions
G1176P141RN11.T0	NcHyBpox1	*N. crassa*	732	Oxidative stress 1 h
G1176P11RL21.T0	NcHyBpox1	*N. crassa*	754	Oxidative stress 1 h
G1176P141FN11.T0	NcHyBpox1	*N. crassa*	730	Oxidative stress 1 h
BG278614.1	NcHyBpox1	*N. crassa*	488	Sexual cDNA library
FG363154	TviHyBpox1	*T. virens*	814	Non-induced mycelium

The 6th clade of hybrid-type B peroxidases comprises dominantly ascomycetous representatives. Few basidiomycetous genes are present too. Most of the genes come from phytopathogenic Sordariomycetes and there is already evidence of their native expression from the EST database (Table 3) mainly in various mycelia grown in minimal media.

**Fig. 8:**
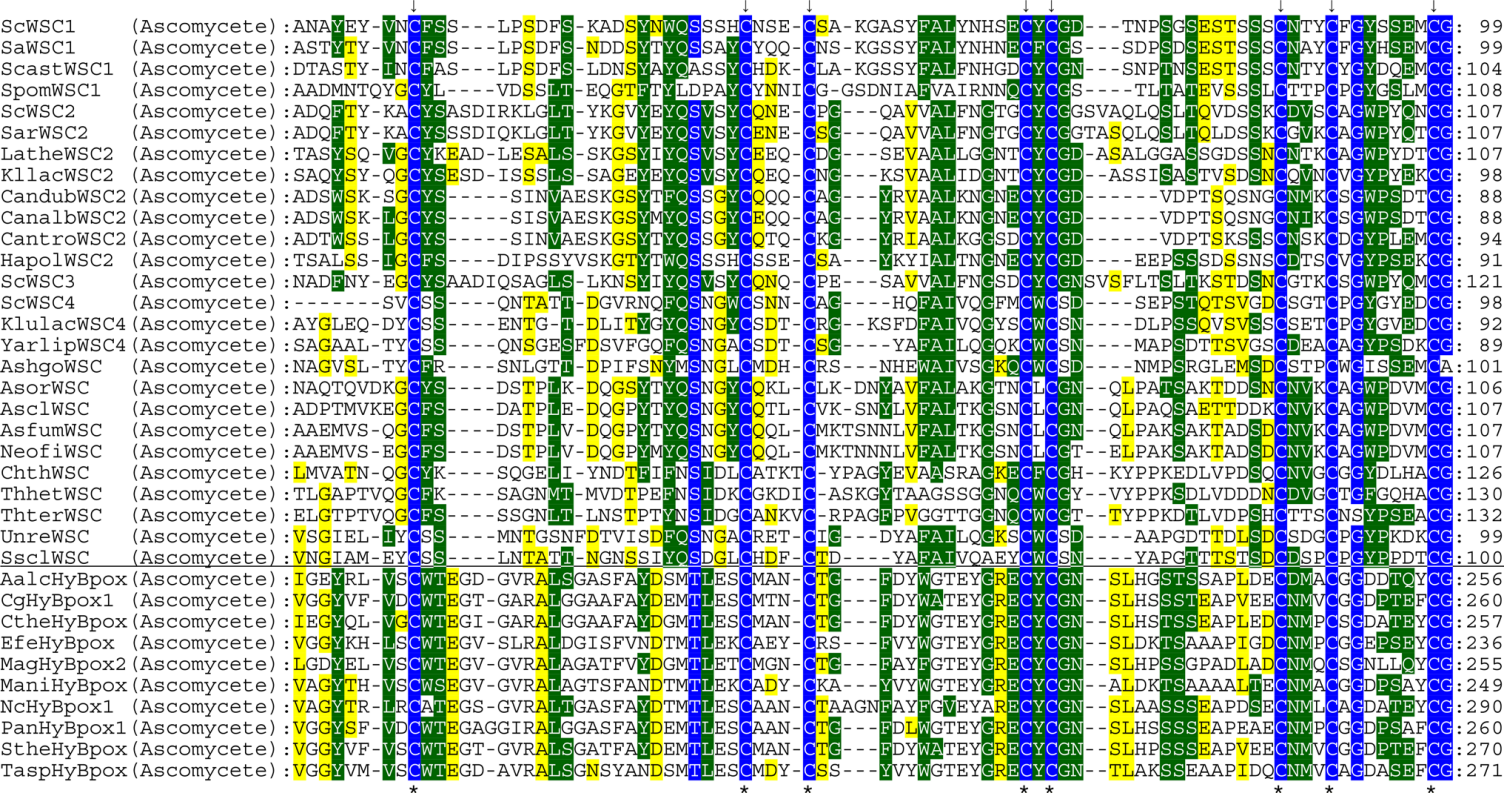
Details of multiple sequence alignment presenting conserved motifs within the WSC domain. The WSC domain of large hybrid-type B peroxidases is compared with that of proteins that do not possess a peroxidase domain. Parameters for the alignment are described in the Sect. “Materials and methods”. Abbreviations of peroxidase names are taken from PeroxiBase

The last clade of the hybrid-type B peroxidase subfamily only contains ascomycetous proteins from Sordariomycetes; among them are also several variants from thermophilic fungi. Expression of peroxidases from this clade was already verified by several EST entries, most prominently in samples induced with oxidative stress (Table 3). The length of their overall open reading frame is at least twice as long as compared to hybrid-type B peroxidases described above due to the presence of a unique C-terminal fusion with WSC domain(s). The latter are classified as PF01822, i.e., a putative carbohydrate binding domain [58]. WSC domain contains up to eight conserved Cys residues that might form several disulfide bridges [59]. It is present only among eukaryotes, mainly in fungal exoglucanases but also among related proteins of higher metazoans. Genetic analysis revealed that WSC proteins are upstream regulators of the stress-activated PKC1-MAP kinase cascade [60]. Figure 8 depicts a multiple sequence alignment of WSC domains in fused hybrid-type B (HyB) peroxidases together with proteins that do not contain a peroxidase domain. A high level of conservation mainly in the region around the eight cysteines is evident.

The physiological function of hybrid-type B peroxidases remains completely unknown. There are currently several EST sequence entries found in non-induced fungi, in fungi induced with oxidative stress and also in fruiting bodies of ascomycetes and basidiomycetes. Sequence analysis suggests that all hybrid-type B peroxidases are secreted (Table 2) carrying a signal peptide of constant length. So far there is no X-ray structure available but in silico analysis predicts intensive N- and O-glycosylation and the occurrence of several disulfide bridges. Structural prediction with I-TASSER suggests that the closest structural homolog of ascomycetous hybrid-type B peroxidase domains is the Class III peroxidase from *Hordeum vulgare* (Table 1). It is important to note that the sequence similarity is rather low.

## Conclusion

The presented phylogenetic reconstruction has demonstrated the important role of hybrid-type heme peroxidases as turning points in the evolution of the complex and abundant peroxidase–catalase superfamily. This computational work provides an excellent basis for detailed spectroscopic and kinetic studies of these missing links and will help to understand the gradual development of structure–function relationships, substrate utilization and, in consequence, the physiological function. Knowledge about structure and catalytic properties will also answer the question whether these proteins are of interest for enzyme engineering and design.

## Abbreviations

APx: Ascorbate peroxidase
APx-CcP: Hybrid-type A peroxidase
CcP: Cytochrome *c* peroxidase
CDE: Chlorite dismutase-dye decolorizing peroxidase-EfeB
EST: Expressed sequence tags
HyBpox: Hybrid-type B peroxidase
KatG: Catalase–peroxidase
PDB: Protein Data Bank
Pfam: Database of protein families
WSC: Cell-wall integrity and stress response component
